# A Course of Practical Histology

**Published:** 1877-07

**Authors:** 


*A Course of Practical Histology.* By EDWARD ALBERT SCHÆFER, Assistant Professor of Physiology in University College, London. With Illustrations. Philadelphia: Henry C. Lea. 1877. 12mo. pp. 304.

To acquire an understanding of the minute anatomical character of a membrane or part, and of the pathological processes which may affect it, is much like the blind learning to read, except one's eyes have the assistance of the microscope, and of printed illustration. With the help of recent works it is not difficult for an ordinary student to recognize with the microscope, the general histological character of tissues and organs, and to verify for himself to considerable extent, the statements of works on medicine or surgery. Histological anatomy, normal and morbid, is in no degree a matter of conjecture, but can be studied practically with advantage, to a certain extent by every earnest student of medicine.

This small volume is an excellent and complete manual for the preparation of microscopic specimens of the various tissues and organs of the body. It has the clearness, simplicity and faithfulness of an experimental investigator, and is on this account attractive and interesting to general students. It is to be used in connection with such descriptive and illustrated works as Frey's Histology and Histo-Chemistry, Stricker's Manual of Histology, and others. The discoid colonies of the blood shown by Osler, are described ; preparations in glycerine are made and recommended more frequently than other authors have done ; and the methods for bringing out different elements of a tissue or organ are fully presented. W.

